# *Platycodon grandiflorus* Polysaccharides Alleviate Cr(VI)-Induced Apoptosis in DF-1 Cells via ROS-Drp1 Signal Pathway

**DOI:** 10.3390/life12122144

**Published:** 2022-12-19

**Authors:** Zhuanglong Zhang, Pimiao Zheng, Changxi Qi, Yuehui Cui, Yijian Qi, Kun Xue, Guangwei Yan, Jianzhu Liu

**Affiliations:** 1 College of Veterinary Medicine, Shandong Agricultural University, Tai’an 271018, China; 2 Research Center for Animal Disease Control Engineering, Shandong Agricultural University, Tai’an 271018, China

**Keywords:** Cr(VI), PGPSt, apoptosis, ROS-Drp1 signal pathway

## Abstract

Hexavalent chromium (Cr(VI)) is a widespread heavy metal that has been identified as a human carcinogen, and acute or chronic exposure to Cr(VI) can cause organ damage. *Platycodon grandiflorus* polysaccharide (PGPS) is a constituent extracted from the Chinese herb *Platycodon grandiflorus*, which has various pharmacological effects. Therefore, the author investigated the role of PGPSt in Cr(VI)-induced apoptosis in chicken embryo fibroblast cell lines (DF-1 cells). Firstly, this study infected DF-1 cells using Cr(VI) to set up a model for cytotoxicity and then added PGPSt. Then, the intracellular reactive oxygen species (ROS), mitochondrial membrane potential (MMP), and apoptosis rate were evaluated. The results showed that PGPSt could inhibit Cr(VI)-induced mitochondrial damage and increase the apoptosis rate. For further exploration of the mechanism of regulation of PGPSt, the ROS-Drp1 pathway was investigated. The antioxidant N-acetyl-L-cysteine (NAC) and mitochondrial division inhibitor 1(Mdivi-1) were added, respectively. The results showed that the NAC and Mdivi-1 restored abnormal mitochondrial fission and cell apoptosis. Thus, PGPSt can alleviate Cr(VI)-induced apoptosis of DF-1 cells through the ROS-Drp1 signaling pathway, which may suggest new research ideas for developing new drugs to alleviate Cr(VI) toxicity.

## 1. Introduction

Hexavalent chromium (Cr(VI)) is a widespread heavy pollutant used in many in-dustries [1]. The industrial use of Cr(VI) waste has gradually increased, with consequent inappropriate disposal. As a result, the environment (soil, water and air) contains Cr(VI), which is a serious threat to human and animal health [2]. Stout et al. [3] demonstrated that exposure to Cr(VI) through the digestive tract resulted in a greater incidence of tumors in mice and rats. Long-term Cr(VI) exposure disturbs the gut microbial homeostasis of chickens [4]. Cr(VI) as a lung carcinogen, confers resistance to thermal stress and interferes with heat shock protein expression in human bronchial epithelial cells [5]. Therefore, Cr(VI) heavy metalloids have been a subject of interest in the area of toxicology. The latest research found that it could cause severe liver and kidney damage [6]. Tang et al. [7] also proved that Cr(VI) could induce hepatocyte apoptosis through the mitochondrial-dependent pathway. Apoptosis is a manner of cells dying under either physiological or pathological conditions. Mitochondrial pathway apoptosis is accompanied by the division of mitochondria, the expression of associated apoptotic proteins, and the formation of apoptotic bodies [8,9]. It is crucial for the body to continue to function properly. There are three pathways through which apoptosis usually occurs, the mitochondrial pathway, the death receptor pathway, and the endoplasmic reticulum (ER) stress pathway [10]. The mitochondrial pathway is the most classical apoptotic pathway. The mitochondria could undergo continuous fission and fusion processes to adapt to changing environments and provide energy to cells [11,12]. Therefore, Mitochondria are the most vulnerable organelles to oxidative stress. Studies have demonstrated that hexavalent chromium can cause excessive accumulation of ROS and lead to mitochondrial dysfunction [13]. Several kinds of literature have reported that Drp1 aggregates the mitochondrial outer membrane under various pressure and interacts with Bax to promote the penetration of pro-apoptotic proteins, including Cyt-c and AIF, into the cytoplasm, triggering mitochondrial fission and apoptosis [14,15]. Plant active ingredients can inhibit Cr(VI)-induced apoptosis. According to existing literature reports, the Astragalus polysaccharide inhibits the radiation-induced apoptosis of megakaryocytes [16]. Angelica polysaccharides can inhibit APAP-induced hepatocyte apoptosis [17].

Our laboratory has found that PGPSt is a very important bioactive component ex-tracted from the Chinese herb Platycodon grandiflorum, which has multiple pharmacological effects including the antioxidation, immunomodulation, anticancer, anti-fatigue, anti-asthma, hepatoprotective and hypoglycemic functions [18]. Xing et al. [19] revealed that PGPSt could inhibit the replication of the pseudorabies virus. Zheng et al. [20] uncovered that PGPSt promotes activated macrophages to boost cellulate immunity. Wang et al. [21] reported that PGPSt could alleviate CCCP-induced apoptosis in 3D4/21 cells. However, whether PGPSt could alleviate Cr(VI)-induced DF-1 cells has not been reported.

In this study, DF-1 cells were subjected to Cr(VI) to establish a cytotoxicity model to investigate the protective effect of PGPSt against Cr(VI)-induced DF-1 cell injury and its mechanism using Cr(VI)-induced mitochondrial-dependent apoptosis. The research lays a particular theoretical foundation for the application of PGPSt in Cr(VI) poisoning. It provides research ideas for the development of new-type drugs to alleviate Cr(VI) poisoning.

## 2. Materials and Methods

### 2.1. Materials and Reagents

In this study, a total PGPSt was prepared by our laboratory [20,22]. K_2_Cr_2_O_7_ was obtained from Kaitong Chemicals (Tianjin Beichen, China). An MMP detection kit (C2006), ROS (DCFH-DA, S0033-1) and a BCA kit (P0012) were obtained from the Beyotime Institute of Biotechnology (Haimen, China). Tubulin antibodies (66240) were purchased from Proteintech (Chicago, IL, USA). A Caspase-3 (cleaved and pro) antibody (31A1067) was provided by Novus (Greenwood Village, CO, USA). A Drp1 (26187) and Bcl-2 antibody (26593) was supplied by Proteintech (Wuhan, China). A Bax antibody (GTX34052) was purchased from Gene Tax (Taft, CA, USA). Annexin V-FITC apoptosis assay kits (A211-01) were purchased from Vazyme (Nanjing, China).

### 2.2. Cell Culture and Treatment

The DF-1 cells were procured from ATCC Agency Company (Beijing, China). DF-1 cells were cultured in a DMEM medium supplemented by 10% serum and 1% penicillin-streptomycin solution at 37 °C in a moistened 5% CO_2_ chamber.

According to previous research in our laboratory [22,23,24], the working concentrations of PGPSt (200 μg/mL) and Cr(VI) (150 μM) was determined. The DF−1 cells were treated with Cr(VI) separately or co−treated with Cr(VI) and PGPSt for 8 h.

### 2.3. Western Blot Analysis

Using RIPA lysis buffer, total cellular proteins were extracted. The cellular sediment was removed, the supernatant was collected and the protein levels were determined using the BCA assay. Proteins were isolated and transferred to a PVDF (polyvinylidene fluoride) membrane via 10% SDS-PAGE gel. Then, the blots were blocked with 5% nonfat milk for 1 h RT and incubated with a primary antibody at 4 °C. Bcl-2 (1:1500), Bax (1:1500), cleaved Caspase 3 (1:1000) and Tubulin (1:1000) were used as primary antibodies. The strips were then incubated with secondary antibodies for 1 h. Goat anti-mouse IgG and goat anti-rabbit IgG antibodies (1:8000) were used as secondary antibodies. Finally, the immunoreactive bands were examined using Enhanced Chemiluminescence (ECL) and the intensity of the immunoreactive bands was analyzed through Image J software.

### 2.4. Measurement of ROS

Reactive oxygen species (ROS) were assessed by stream cytometry using the flu-orescent stain DCFH-DA. Briefly, after the treatment of cells, DF-1 cells were harvested and incubated with DCFH-DA at 37 °C for 20 min and residual probes were laundered with serum-free medium and detected by flow cytometry.

### 2.5. Measurement of MMP

The mitochondrial membrane potential (MMP) was calculated by stream cytometry using the fluorescent stain JC-1. The reaction principle was that: when the MMP value was high, JC-1 aggregated in the mitochondrial matrix to form a polymer that emitted red fluorescence; when the MMP value was low, JC-1 was the monomer that had a green fluorescence. The DF-1 cells were collected and incubated with JC-1 dye for 20 min at 37 °C. The remaining unreacted reagents were removed by JC-1 washing buffer. Finally, the resulting fluorescence was detected by flow cytometry.

### 2.6. Annexin V-FITC/PI Staining for Apoptosis Detection

Apoptosis rates were estimated by stream cytometry using the fluorescent dye Annex-in V-FITC and PI. Briefly, DF-1 cells were collected and washed twice with prechilled PBS. Finally, batteries were spiked in 100 μL of binding buffer, incubated with Annexin V-FITC and PI for 15 min, and then 400 μL of binding buffer was added. Detection was carried out by flow cytometry.

### 2.7. Statistical Analysis

Statistical analysis was performed using GraphPad Prism Version 8.0. Data were obtained from at least three separately performed experiments and were expressed as mean ± SD. Differences between groups were analyzed using ANOVA and Student’s *t*-test. Statistically significant differences were assumed at *p* < 0.05.

## 3. Results

### 3.1. PGPSt Alleviated Cr(VI)-Induced Mitochondrial Damage

**Figure 1:**
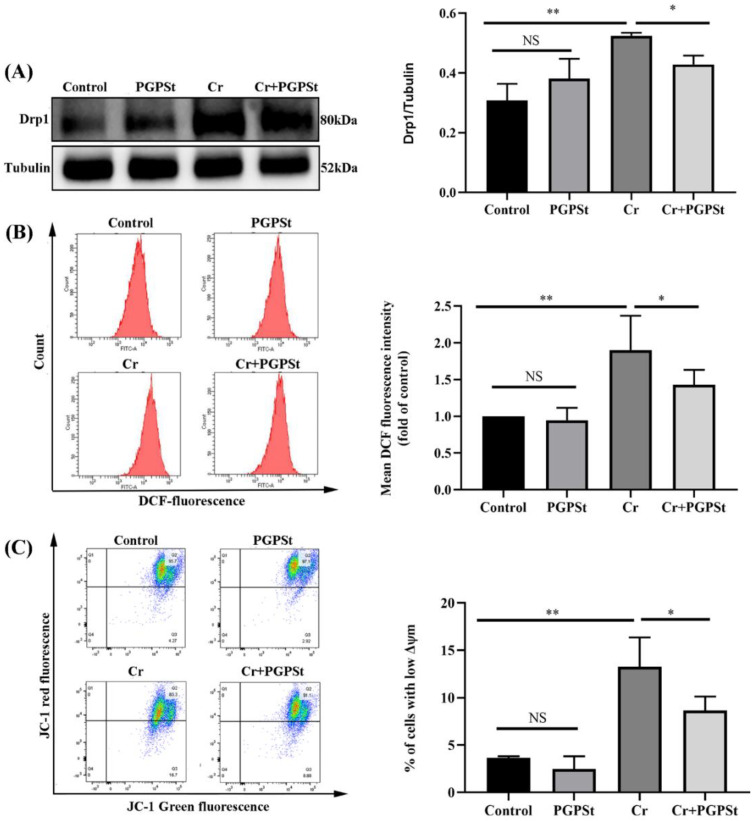
**PGPS_t_ alleviated Cr(VI)-induced mitochondrial damage.** (**A**) Detection of Drp1 protein by Western blot and quantitative data analysis of Drp1 protein. (**B**) ROS detection by flow cytometry and statistical analysis of ROS results. (**C**) Quadrant plot of MMP detection by flow cytometry and statistical analysis of the results. Experimental data results are expressed as mean ± SD (n = 3). * *p* < 0.05, indicating a significant difference. ** *p* < 0.01, indicating an extremely significant difference. NS *p* > 0.05, indicates that the difference is not significant.

To study the effect of PGPSt on alleviating Cr(VI)-induced apoptosis of DF-1 cells, the oxidative stress and mitochondrial damage state were examined. DF-1 cells were pretreated with 150 μM Cr(VI) for 8 h before other experiments. It is known that the Drp1 protein is the critical protein causing mitochondrial fission [25]. The research tested the expression of the Drp1 protein by Western blot to prove that Cr(VI) could cause mitochondrial division. From Figure 1A, the expression of Drp1 protein was significantly increased in the Cr(VI)-treated group, leading to the migration of Drp1 to the mitochondria. PGPSt significantly inhibited the increase of the Drp1 protein level compared to the Cr(VI) treated group. In addition, some reports [15,26]. proved that the activation of Drp1, especially its translocation, depended on ROS, so we measured the ROS levels. As shown in Figure 1B, the ROS levels detected by stream cytometry demonstrated a significant increase in ROS for the 150 μM Cr(VI)-treated group compared to the control group. In contrast, the ROS levels decreased after PGPSt treatment. The decreased MMP was a biomarker of mitochondrial damage. In general, the extent of cellular damage increases as the MMP decreases. Compared with the control group, the MMP was significantly decreased in the Cr(VI) group, and the MMP was significantly increased in the PGPSt group (shown in Figure 1C). These results suggested that Cr(VI) induced mitochondrial damage in DF-1 cells, whereas PGPSt could significantly attenuate mitochondrial damage.

### 3.2. Inhibition of Cr(VI) Induction of Mitochondria-Dependent Apoptosis by PGPSt

**Figure 2:**
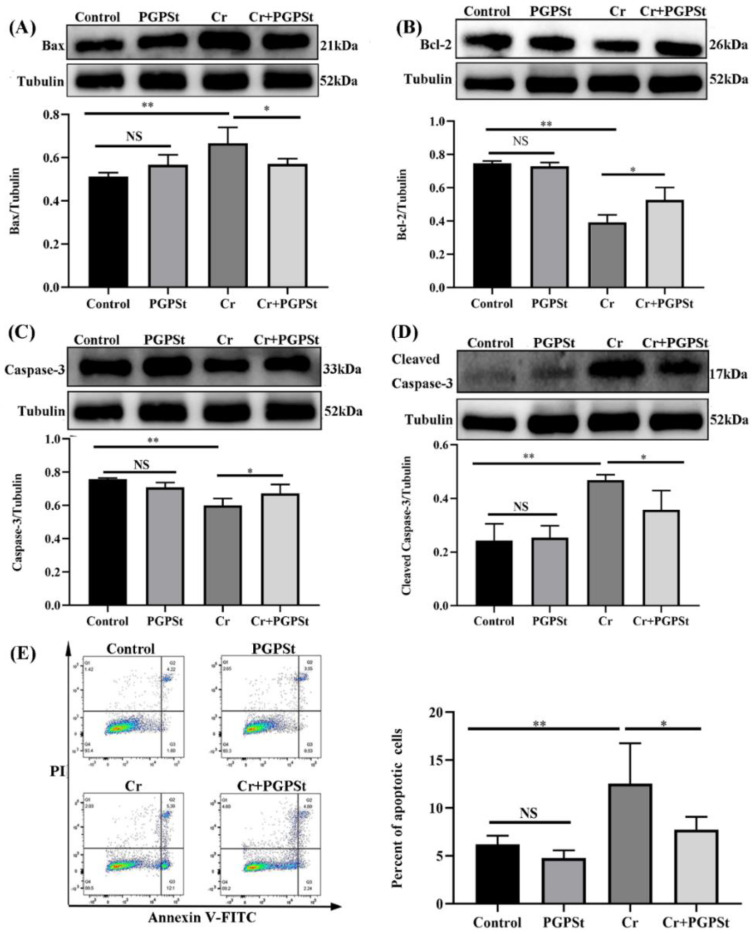
**PGPS_t_ suppressed Cr(VI)-induced mitochondrial-dependent apoptosis.** (**A**) Detection of Bax protein by Western blot and quantitative data analysis of Bax protein. (**B**) Detection of Bcl-2 protein by Western blot and quantitative data analysis of Bcl-2 protein. (**C**) Detection of Caspase-3 protein by Western blot and quantitative data analysis of Caspase-3. (**D**) Detection of Cleaved Caspase-3 protein by Western blot and quantitative data analysis of Cleaved Caspase-3 protein. (**E**) Quadrant plot of apoptosis rate detected by flow cytometry and statistical analysis of results. Experimental data results are expressed as mean ± SD (n = 3). * *p* < 0.05, indicating a significant difference. ** *p* < 0.01, indicating an extremely significant difference. NS *p* > 0.05, indicates that the difference is not significant.

For explaining the inhibition of Cr(VI)-induced mitochondrial-dependent apoptosis by PGPSt, the research examined the relevant apoptotic proteins and the cell apoptotic rate. The Bcl-2 family of apoptosis-related proteins is a pivotal modulator of apoptosis, and its anti-apoptotic and pro-apoptotic members work together to act as an apoptosis switch [27]. From Figure 2A, the expression of Bax in Cr(VI)-treated cells increased significantly. PGPSt (200 μg/mL) significantly inhibited the increase of the Bax protein level compared with the Cr(VI) group. From Figure 2B, compared with the control group, the Bcl-2 protein expression was decreased in the Cr(VI)-treated group. PGPSt could alleviate the above changes. As shown in Figure 2C, there was a significant decrease in Caspase-3 protein expression after Cr(VI) treatment and an increase in Caspase-3 protein level after PGPSt with Cr(VI) co-treatment. From Figure 2D, due to the significant increase of Cleaved Caspase-3 levels in the Cr(VI) treated group, PGPSt (200 μg/mL) significantly inhibited the increase of Cleaved Caspase-3 protein level compared with the Cr(VI) treated group. The detection of the apoptosis rate of DF-1 cells by flow cytometry is shown in Figure 2E. The apoptosis rate was significantly higher in the Cr(VI)-treated group compared to the control group. However, this change was alleviated by PGPSt treatment. These results showed that PGPSt could significantly inhibit the Cr(VI)-induced apoptosis rate in chicken embryo fibroblast cell lines.

### 3.3. Effect of NAC on Mitochondrial Damage and Cellular Apoptosis in Cr(VI)-Exposed DF-1 Cells

**Figure 3:**
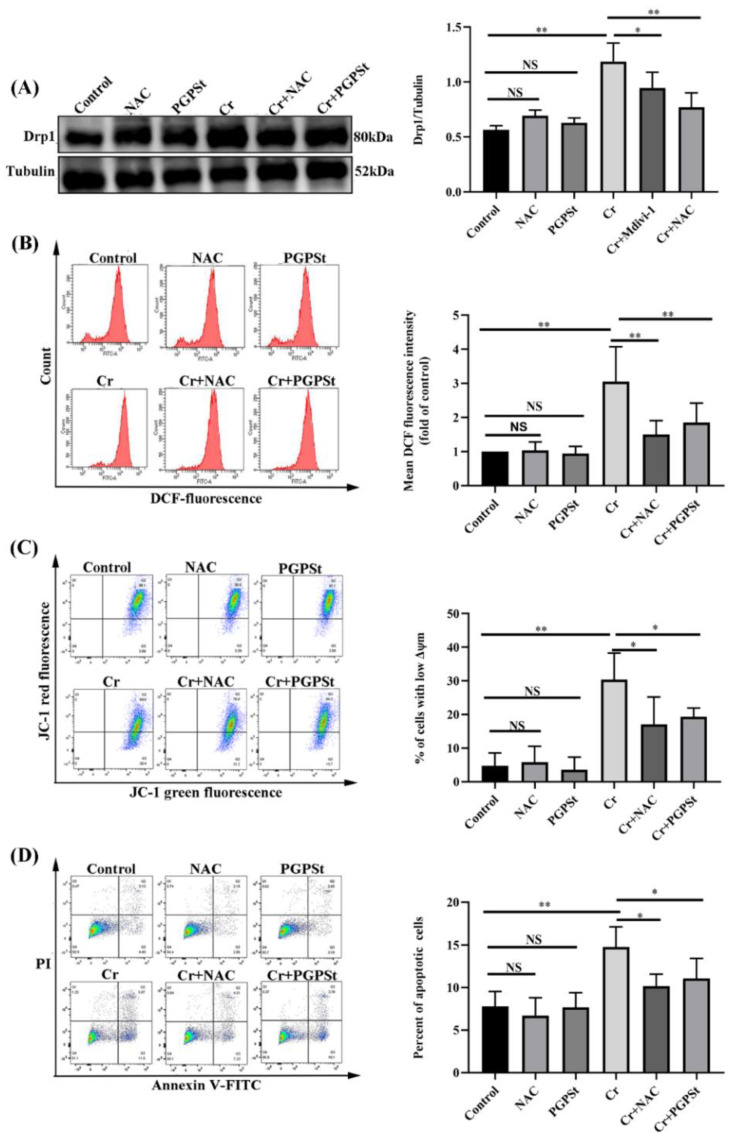
**Effect of NAC on mitochondrial damage and cell apoptosis in Cr(VI)-exposed DF-1 cells**. (**A**) Detection of Drp1 protein by Western blot and quantitative data analysis of Drp1 protein. (**B**) ROS detection by flow cytometry and statistical analysis of ROS results. (**C**) Quadrant plot of MMP detection by flow cytometry and statistical analysis of the results. (**D**) Quadrant plot of apoptosis rate detected by flow cytometry and statistical analysis of results. Experimental data results are expressed as mean ± SD (n = 3). * *p* < 0.05, indicating a significant difference. ** *p* < 0.01, indicating an extremely significant difference. NS *p* > 0.05, indicates that the difference is not significant.

For confirming the possibility that excessive accumulation of ROS is associated with Cr(VI)-mediated mitochondrial-dependent apoptosis, the study used an inhibitor (antioxidant N-acetyl-L-cysteine, NAC). The Cr(VI)-treated group caused up-regulation of Drp1 protein compared with the control group, as shown in Figure 3A. The results showed that NAC and PGPSt treatment of cells significantly inhibited the increase in Drp1 expression. Detection of ROS content by stream cytometry demonstrated that the level of ROS in the 150 μM Cr(VI)-treated group was remarkably increased more than in the control group (Figure 3B). NAC and PGPSt treatment markedly inhibited Cr(VI)-induced ROS formation. Cr(VI)-induced ROS formation was significantly inhibited by NAC and PGPSt. Flow cytometry was used for analysis, and the 150 μM Cr(VI)-treated group had a significantly greater decrease in MMP than the control group, as shown in Figure 3C. NAC and PGPSt treatment significantly suppressed the decrease of MMP. As shown in Figure 3D, the Cr(VI) group apoptosis rate detected by stream cytometry was dramatically higher than the control group, but this change was alleviated by NAC and PGPSt treatment. The obvious protective effect of NAC indicated that oxidative stress was closely related to Cr(VI)-induced mitochondrial dysfunction and apoptosis. PGPSt could attenuate apoptosis by scavenging ROS.

### 3.4. Effect of Midivi-1 on Mitochondrial Damage and Cellular Apoptosis in Cr(VI)-Exposed DF-1 Cells

**Figure 4:**
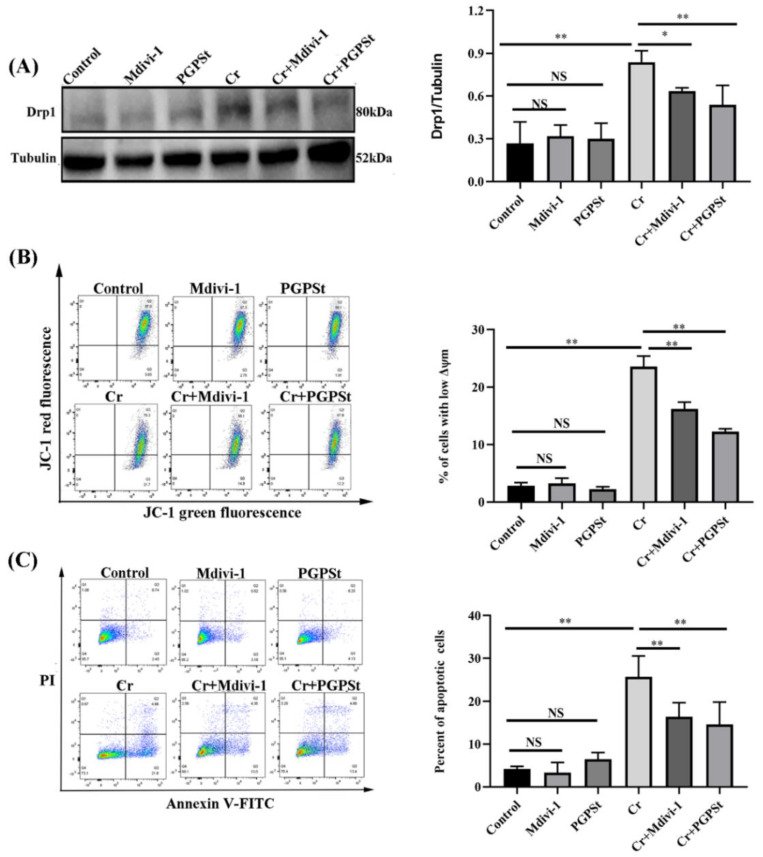
**Effect of midivi-1 on mitochondrial damage and cell apoptosis in Cr(VI)-exposed DF-1 cells.** (**A**) Detection of Drp1 protein by Western blot and quantitative data analysis of Drp1 protein. (**B**) Quadrant plot of MMP detection by flow cytometry and statistical analysis of the results. (**C**) Quadrant plot of apoptosis rate detected by flow cytometry and statistical analysis of results. Experimental data results are expressed as mean ± SD (n = 3). * *p* < 0.05, indicating a significant difference. ** *p* < 0.01, indicating an extremely significant difference. NS *p* > 0.05, indicates that the difference is not significant.

A mitochondrial fission inhibitor 1 (Mdivi-1) could effectively inhibit mitochondrial fission, so it was used to evaluate whether the mitochondrial fission state is associated with apoptosis. Figure 4A, Cr(VI) treatment caused up-regulation of Drp1 protein compared to the control group. Mdivi-1 and PGPSt treatment significantly suppressed the increase of Drp1 expression. In Figure 4B, the flow-through results showed that the lower MMP in the 150 μM Cr(VI)-treated group was significantly higher than the control group. Mdivi-1 and PGPSt treatment significantly inhibited the reduction of MMP. As shown in Figure 4C, flow cytometry analysis revealed that the apoptosis rate was significantly higher in the 150 μM Cr(VI)-treated group than in the control group. The situation was resolved after treatment with PGPSt and Mdivi-1. These data suggest that PGPSt arrests Cr(VI)-induced mitochondria-dependent apoptosis by inhibiting mitochondrial fission.

## 4. Discussion

Improper handling of illegally discharged toxic chromium during industrial pro-duction has become a worldwide problem of environmental pollution [28]. Cr(VI) is recognized as the most poisonous version of chromium and can cause severe damage to the body. Cr(VI) also can enter cells through the cell membrane, causing mitochondrial damage, DNA chain rupture and even other toxic effects [29]. Ge et al. [30] clarified that Cr(VI)induced apoptosis in A549 cells through endoplasmic reticulum stress and Liang et al. [31] certified that Cr (VI) induced mitochondria-dependent apoptosis in L02 hepatocytes. However, there are few reports on Cr (VI)-induced apoptosis in DF-1 cells. This study revealed for the first time the role of PGPSt in Cr(VI)-induced apoptosis in DF-1 cells. As dynamic organelles, mitochondria engage in ongoing division and fission. Alterations in mitochondrial form and dynamics have been reported to obviously influence nearly the entire spectrum of mitochondrial function and are associated with the evolution of various diseases [32]. The relationship between the pathway apoptosis associated with the mitochondrial division process in DF-1 cells exposed to Cr(VI) is not completely clear. Previous studies have indicated that Cr(VI)-induced mitochondrial damage is characterized by the accumulation of intracellular reactive oxygen species, which could simultaneously reduce mitochondrial membrane potential and increase Drp1 protein expression. Mitochondrial dysfunction could lead to the release of apoptotic factors to promote apoptosis [33].

As shown in Figure 2, Cr(VI) could lead to elevated expression of Bax, cleaved Caspase-3 protein, and decreased expression of Bcl-2 protein, resulting in an increased apoptosis rate. These results suggested that mitochondrial dysfunction and altered mi-tochondrial morphology were an important role in promoting Cr(VI)-induced apoptosis in DF-1 cells. Mitochondria are the primary source of ROS and the most vulnerable organelle [34,35]. This study investigated that mitochondrial pathway apoptosis induced by Cr(VI) was accompanied by the accumulation of ROS. And the research that NAC as the ROS scavenger was used to investigate the role of ROS in Cr(VI)-induced mitochondrial pathway apoptosis. Indeed, the treatment with NAC significantly inhibited the accumulation of ROS and decreased the expression of Drp1 protein and increased the MMP content in the Cr(VI)-treated group. Interestingly, NAC almost completely reversed Cr(VI)-induced apoptosis. Furthermore, this experiment demonstrates that ROS is an upstream regulator of Drp1 transport and activation, and its presence could increase the expression of Drp1 in mitochondria. Subsequently, we applied Mdivi-1 to study the role of Drp1 in Cr(VI)-induced mitochondrial pathway apoptosis. The results revealed that Mdivi-1 treatment considerably inhibited mitochondrial dysfunction and apoptosis. The above results suggested that Cr(VI) can induce apoptosis of DF-1 cells through the ROS-Drp1 signaling pathway.

PGPSt is one of the substantial bioactive components extracted from the traditional Chinese herb Platycodon, which is increasingly used due to its low side effects, antioxidant and other pharmacological effects [36]. This study investigated the activity of PGPSt in Cr(VI)-induced mitochondrial pathway apoptosis in DF-1 cells. The results illustrated that PGPSt could inhibit the accumulation of ROS, decrease the expression of Drp1 protein and alleviate mitochondrial damage, thereby inhibiting the expression of related apoptotic proteins and reducing the apoptosis rate of cells. This study demonstrates for the first time that PGPSt inhibits Cr(VI)-mediated apoptosis in DF-1 cells through the ROS-Drp1 signal pathway, thus confirming the protective mechanism of PGPSt against mitochondrial-dependent apoptosis-mediated DF-1 cell injury.

## 5. Conclusions

This study investigated the activity of PGPSt in Cr(VI)-induced apoptosis in chicken embryo fibroblast cell lines (DF-1 cells). In conclusion, Cr (VI) induced the accumulation of ROS and induced mitochondrial division, which led to DF-1 apoptosis. PGPSt (200 μg/mL) can regulate ROS and mitochondrial division and alleviate apoptosis through the ROS-Drp1 pathway. Therefore, as a potential drug, PGPSt could mitigate Cr(VI)-induced DF-1 cell destruction.
